# Iodine-125-labeled cRGD-gold nanoparticles as tumor-targeted radiosensitizer and imaging agent

**DOI:** 10.1186/s11671-015-0864-9

**Published:** 2015-04-02

**Authors:** Ning Su, Yajie Dang, Guangli Liang, Guizhi Liu

**Affiliations:** Department of Radiation Oncology, Tianjin Medical University Cancer Institute and Hospital, National Clinical Research Center of Cancer, Key Laboratory of Cancer Prevention and Therapy, Tianjin, 300060 China; Department of Respiratory Medicine, First People’s Hospital Affiliated to Shanghai Jiao Tong University, Shanghai, 201600 China

**Keywords:** Radiotherapy, Radiosensitizer, cRGD-gold nanoparticles, Iodine-125, Apoptosis

## Abstract

Research interests on radiosensitive property of gold nanoparticles (GNPs) are rapidly raised because of the extensively proved *in vitro* effectiveness and clinical necessity. However, the issue of targeted accumulation of GNPs in tumor tissues hindered the transference to *in vivo* applications. In this study, hybrid nano-sized cyclic Arg-Gly-Asp-conjugated GNPs (cRGD-GNPs) integrated with radioactive iodine-125 was fabricated as tumor-targeted radiosensitizer. Therapeutic effects, including acute apoptosis (2 days post treatment) and long-term influence (up to 21 days), were investigated on NCI-H446 tumor-bearing mice via Tc-99 m-Annexin V SPECT and volume measurements, respectively. Apoptosis and volume loss were consistent in showing that tumor growth was effectively suppressed via the treatment of ^125^I-cRGD-GNP sensitized radiotherapy (RT), a more significantly radiosensitive effect than the treatment of non-targeted GNPs with RT, RT treatment alone, and no treatment. SPECT/CT images showed that the uptake of cRGD-GNPs by tumor tissues reached the peak target/non-target value of 4.76 at around 2 h post injection, and dynamic radioactivity monitoring showed that ^125^I-cRGD-GNPs maintained about 2.5% of injected dosage at 55 h post injection. For long-term influence, a significant radiosensitized RT-induced volume loss was observed. Hence, cyclic RGD conjugation makes the GNP-based radiosensitizer tumor targeting, offering a new modality for enhancing radiotherapeutic efficacy. Additionally, the introduction of I-125 serves as both a therapeutic factor and a radiotracer for *in vivo* tracking of GNPs.

## Background

Surface plasmon resonance and the ability to bind thiol and amine group enabled the biological and medical applications of gold nanoparticles (GNPs) [1], such as photodynamic therapy [2], therapeutic delivery agents [3], diagnostic agents [4,5], and radiosensitizer [6,7]. Remarkably, research on radiosensitive properties of gold nanoparticles and corresponding mechanism has got a lot of progress. In general, dose enhancement factor (DEF) related to radiosensitive effect ranges from 1.01 to 2.11 [8], depending on various items, such as the dosage of radiation, concentration, size, morphology, surface coating, and charging of radiosensitizer. For radiation energy, kV radiotherapy (RT) carries higher DEF than MV energy [9] but is limited to superficial tumors because of the weak penetrability. For tumor targeting, GNPs may passively accumulate in tumor tissues due to enhanced permeability and retention (EPR) effect of immature blood vessels, but target molecules, such as antibodies, could promote active targeting process, where an interaction of cell membrane and target molecules on the surface existed. On the other side, size and morphology influence the cellular uptake and cytotoxicity. As a general rule, a diameter of less than 30 nm is deemed as necessary for adequate cellular import [10], but smaller GNPs bring bigger toxicity [11,12]. The uptake of shorter rod-shaped GNPs is higher than that of longer ones, but both are lower than spherical ones [13]. On the contrary, rod-shaped GNPs were exocytosed faster than spherical ones because of the relatively less conjugated binding factors [14]. For *in vivo* applications, besides EPR effect, reticuloendothelial system (RES) also heavily influences the *in vivo* distribution of nano-sized radiosensitizer. Smaller size is helpful in avoiding the RES uptake [15]. Moreover, polyethylene glycol (PEG) coating is effective to reduce the RES uptake and increase circulation times, leading to a significant growth of GNP amount in tumor (10- to 100-folds, compared to nude GNPs) [16,17] and less spleen and liver damage [18]. Hence, compromises among these items above are considerable for practical applications.

Radiosensitive property, reflected as drug efficacy, is heavily influenced by targeting efficiency. The αβ serial integrins in regulation of angiogenesis is one of the most promising and best studied targets in oncology research [19]. Correspondingly, Arg-Gly-Asp (RGD)-based strategy to target α_v_β_3_ integrin, which mediates various cancer stages, such as malignant transformation, tumor growth and progression, invasion, and metastasis, is most researched in cancer diagnosis and therapy. Conjugation of RGD peptides enables drugs to recognize α_v_β_3_ integrin, abundantly expressed on tumor blood vessels but not on vessels of normal tissues [20]. Therefore, lots of RGD-based α_v_β_3_-targeted drugs, including hybrid nanoparticles, have been designed as therapy drug, drug delivery system, imaging agent, and complex functionalized drugs. Chemotherapeutics, such as doxorubicin, has been loaded by RGD-based nanoparticles [21]; moreover, the more complex ‘all-in-one’ RGD-based magnetic nanoparticles, which contained iron oxides as contrast agent, siRNA as therapeutic agent, and a fluorescent dye for fluorescent microscopy, were designed and evaluated *in vitro* [22]. Particularly, RGD-conjugated gold nanoparticles were reported and tested for targeting efficiency and radiosensitization as well, but *in vitro* evaluation was rarely reported [23]. Overall, the targeting of RGD-grafted nanoparticles to tumoral endothelium was more effective than non-targeted nanoparticles [24,25]. Additionally, safety profiles and no-dose-limiting toxicity proved in patients are more advantaged than antibody strategies [26,27]. Hence, RGD-based strategy provides a promising and efficient approach in guiding the GNPs.

Herein, we chose the 20-nm GNPs as the platform for constructing the multifunctional agent. Cyclic RGDyC peptides were chosen considering the rigidity and variety of chemical modification. In order to evaluate the *in vivo* targeting efficiency and RT effect of the targeting radiosensitizer, human small-cell lung cancer model, NCI-H446 tumor cell-bearing mice model, was utilized due to the relatively moderate sensitivity to RT and the clinical needs. For the assessment of RT effect, Tc-99 m-Annexin V SPECT was utilized in early evaluation of apoptosis induced by radiosensitized RT for the first time and cooperated with tumor volume measurements, guaranteeing the consistency of acute apoptosis assessment and long-term observation of therapeutic effect. In this study, radionuclide was also introduced in fabrication of radiosensitizer. Iodine-125 was labeled to the cRGD-GNPs and delivered an effective radiation dose to tumor cells with minimal damage to normal tissues [28]. As a clinically used therapeutic radionuclide, I-125 contributes to the radiotherapy effect; even more importantly, I-125 provides an approach of *in vivo* dynamic monitoring to figure out biodistributions of cRGD-GNPs.

## Methods

### Materials and instruments

The following instruments were used: cobalt-60 radioactive point source, NanoSPECT/CT (Bioscan, Washington, DC, USA); radioactivity meter (CRC-25R, Capintec Inc., Ramsey, NJ, USA); centrifuge (80–1, Zhengzhou Boke Instrument Equipment Co., Ltd., Henan, China); transmission electron microscope (TEM; 2100 F, JEOL Ltd., Tokyo, Japan); and zetasizer Nano (ZSP, Malvern Instruments Ltd., Worcestershire, UK).

The following materials and reagents were used: 1,3,4,6-tetrachloro-3a,6a-diphenylglucoluril (Iodogen) and 4-(N-maleimidomethyl)cyclohexane-1-carboxylic acid 3-sulfo-N-hydroxysuccinimide ester sodium salt (Sulfo-SMCC) were purchased from Sigma-Aldrich, St. Louis, MI, USA. Additional materials include Annexin V (human recombinant, Abcam, Cambridge, MA, USA), cyclic RGDyC peptide (cyclo (Arg-Gly-Asp-Tyr-Cys), GL Biochem Ltd., Shanghai, China); Na^99m^TcO_4_ and Na^125^I (China Isotope & Radiation Corporation, Beijing, China); Whatman 3MM chromatography paper (GE Healthcare UK Limited, Buckinghamshire, UK), and BD Matrigel™ Basement Membrane Matrix. Diethylene triamine pentacetate acid (DTPA), amine-PEG-thiol (*M*_W_ = 3 kDa, 1,500 equivalents), analytical phosphate-buffered saline (PBS; 0.2 M), SnCl_2_ · 2H_2_O, and acetone were purchased from China National Pharmaceutical Group Corporation, Beijing, China. ^99m^Tc-DTPA-Annexin V was prepared in-house following the reported method with radiochemical purity >95% [29].

### Animal models

All animal experiments were approved by the Tianjin Medical University Cancer Institute and Hospital Animal Care Committee and were in accordance with the ethical guidelines of the National Institutes of Health. All efforts were made to minimize animal suffering and the number of animals that were used. Twenty BALB/C mice (male, 10 weeks, weight of 20 ± 1 g each) were provided by the Institute of Laboratory Animal Science, CAMS. Mice were raised in a SPF class experimental animal room. 5 × 10^6^ cells (in 30 μL Matrigel) were subcutaneously injected into the right hind leg to prepare the NCI-H466 tumor model mice. Tumor-bearing mice were ready for treatment when tumor volume reached 80 to 100 mm^3^ (10 to 14 days needed).

### Synthesis and characterization of ^125^I-cRGD-GNPs

**Figure 1:**
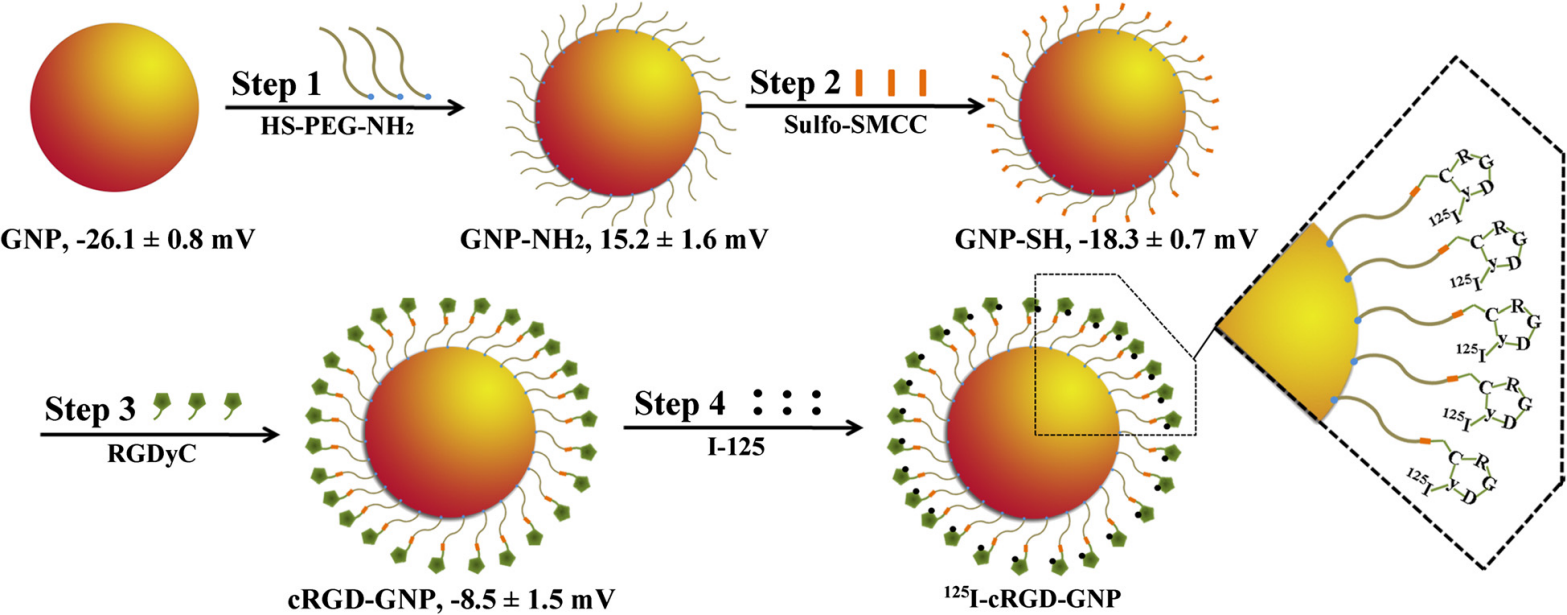
**Schematic synthesis procedure of** ^**125**^ **I-cRGD-GNPs and changes of surface ζ potential.** The schematic procedure of hybrid nanoparticles includes PEG covering, surface modification, cyclic RGD conjugation, and I-125 labeling.

In a three-necked flask with reflux unit, HAuCl_4_ was dissolved in 50 mL distilled water at a concentration of 0.1 mg/mL and then heated to 100°C. One milliliter of 10 mg/mL sodium citrate solution was added, and the reaction lasted for another 5 min. The reaction system was naturally cooled down to room temperature, and the product was collected with a 10,000 nominal molecular weight limit centrifugal filter units. Gold nanoparticles were stored at a concentration of 1 mg/mL. Surface modification followed the reported procedures with some improvements [25,30]: (i) GNPs were pre-treated with Tween 20. Tween 20 (0.1 mL) was added to 10 mL of GNPs and then stirred for 10 min. (ii) After the addition of 1 mL amine-PEG-thiol, the mixture was stirred for 2 h to allow complete binding between GNPs and thiol groups. The mixture was purified by centrifugation twice (10,000 rpm for 10 min, at 4°C, hereinafter the same below). The product was redispersed in 10 mL HEPES buffer (0.3 M, pH = 8.0). (iii) Sulfo-SMCC was linked to the amine part of PEG-coated GNPs. Freshly dissolved sulfo-SMCC (1 mL, 100 μM, in DMSO) was added to the suspension above. The mixture was vortexed for 1 h and then purified by centrifugation twice. (iv) For cyclic RGDyC peptide conjugation, the supernatant was redispersed in 10 mL of cyclic RGDyC peptide (100 μM) in PBS (pH = 7.4) and then vortexed for 1 h. cRGD-GNPs was harvested after centrifugation and kept at 4°C and in dark condition for long-term storage. (v) Iodine labeling was performed before the *in vivo* applications. Na^125^I (370 MBq) and iodogen (20 μg) were added into the above cRGD-GNP suspension (containing 10-mg GNPs). The reaction lasted for 10 min at room temperature. The schematic synthesis procedure is described in Figure 1.

The morphology and size of GNPs were characterized using TEM at an acceleration voltage of 200 kV; UV absorbance of nude GNPs was measured to detect maximum absorption peak, and UV spectrums of liquid supernatant of cRGD conjugation reaction system were obtained before and after the reaction to confirm the conjugation of cRGD. The size distribution of PEG-coated GNPs was measured by dynamic light scattering (DLS); measurement of surface *ζ* potential was performed by zetasizer to evaluate the PEG covering and cRGD coupling. The *in vitro* stability of ^125^I-cRGD-GNPs was tested by incubating ^125^I-cRGD-GNPs in 0.05 M PBS or 10% fetal bovine serum (FBS) for 3 days at 37°C. Radiolabeling efficiency and radio-chemical purity were monitored by thin-layer chromatography silica-gel-based thin-layer chromatography with 0.9% NaCl as developing solvent: *R*_f_ of ^125^I-cRGD-GNPs is 0 and *R*_f_ of I-125 is 1.

### Investigation of biodistribution

I-125-labeled cRGD-GNPs (37 MBq, containing 1-mg GNPs) were injected intravenously. At 1, 2, and 4 h post injection, mice were anesthetized via 2% isoflurane inhalation. SPECT was performed with the following parameters: four high-resolution, conical collimators with nine-pinhole plates; energy peak, 28 keV; window width, 10%; resolution, 1 mm/pixel; matrix, 256 × 256; and scan time, 60 s/projection with 24 projections in all. Three-dimensional ordered subset expectation maximization images were reconstructed using HiSPECT (Bioscan, Washington D.C., USA). CT was then performed with the following scan parameters: frame resolution, 256 × 512; tube voltage, 45 kVp; current, 0.15 mA; and exposure time, 500 ms/frame. Real-time 3D reconstructions of the collected images were performed using the Nucline software (v 1.02, Mediso, Hungary). A fused SPECT/CT image was generated for investigation of biodistribution.

Drug residual was inspected by the radioactivity meter, and the radioactivity of excreta was recorded by the *γ* counter as well to detect the metabolic pathways. A value lower than 2.5% of the injected dosage was deemed as roughly cleared. Samples of blood, urine, and feces were collected immediately after injection and at 0.5, 1, 2, 4, and 10 h and 1, 2, and 3 days post injection and then measured by the *γ* counter to investigate the %ID/g. All the recorded data of radioactivity was corrected to the original injected radioactivity dosage.

### Radiotherapy of tumor-bearing mice

Twenty NCI-H466 tumor-bearing mice were divided into five groups for treatments: ^125^I-cRGD-GNPs with radiotherapy, cRGD-GNPs with radiotherapy, GNPs with radiotherapy, radiotherapy alone, and control group (no treatment). Injections were sterilized by 0.22-μm filter membrane. For the radiosensitized radiotherapy, 100 μL (containing 1-mg Au) ^125^I-cRGD-GNPs, cRGD-GNPs, or GNPs were injected intravenously. At the definite time, mice were anesthetized via intraperitoneal injection of 150 μL 4% chloral hydrate. Tumor tissues were exposed to 5-Gy *γ* rays emitted by the Co-60 source. The injected doses were normalized to about 50-μg GNPs every gram body weight.

### Investigation of therapeutic effect

Two days after treatment, ^99m^Tc-Annexin V (18.5 MBq/mouse) was administered intravenously for apoptosis evaluation. At 2 h after the injection, mice were anesthetized via 2% isoflurane inhalation. SPECT was performed using the following parameters: four high-resolution, conical collimators with nine-pinhole plates; energy peak, 140 keV; window width, 10%; resolution, 1 mm/pixel; matrix, 256 × 256; and scan time, 60 s/projection with 24 projections in all. Target/non-target (T/NT) analysis focused on the treated tumor tissues and corresponding comparison to the tissues on the left leg.

For assessments of long-term influence, tumor sizes were measured every 3 days (during early stage) or every 6 days (during later stage) and calculated as follows: Volume = (Tumor length) × (Tumor width)^2^/2. The body weight of every mouse was recorded as well. Mice were sacrificed at the 21st day after treatment, and the weight of excised tumors was recorded.

### Statistics

Data of volume and weight were expressed as mean ± standard deviation (SD). One-way (treatment method) analysis of variance (ANOVA) followed by *post hoc* test was used to analyze the differences between groups. Statistics were performed in SPSS 19.0.

## Results and discussion

### Synthesis of ^125^I-cRGD-GNPs

The as-synthesized GNPs were of a uniform size of 21.7 ± 2.1 nm, and the maximum UV absorption peak was at 537 nm. DLS hydrodynamic diameter of PEG-covered GNPs was 45.2 ± 2.6 nm. cRGD was successfully conjugated to GNPs based on the changes of surface *ζ* potential and RGD concentration changes of reaction supernate reflected by the UV spectrum. The surface *ζ* potentials of ^125^I-cRGD-GNP synthesis after every step are marked in Figure 1.

**Figure 2:**
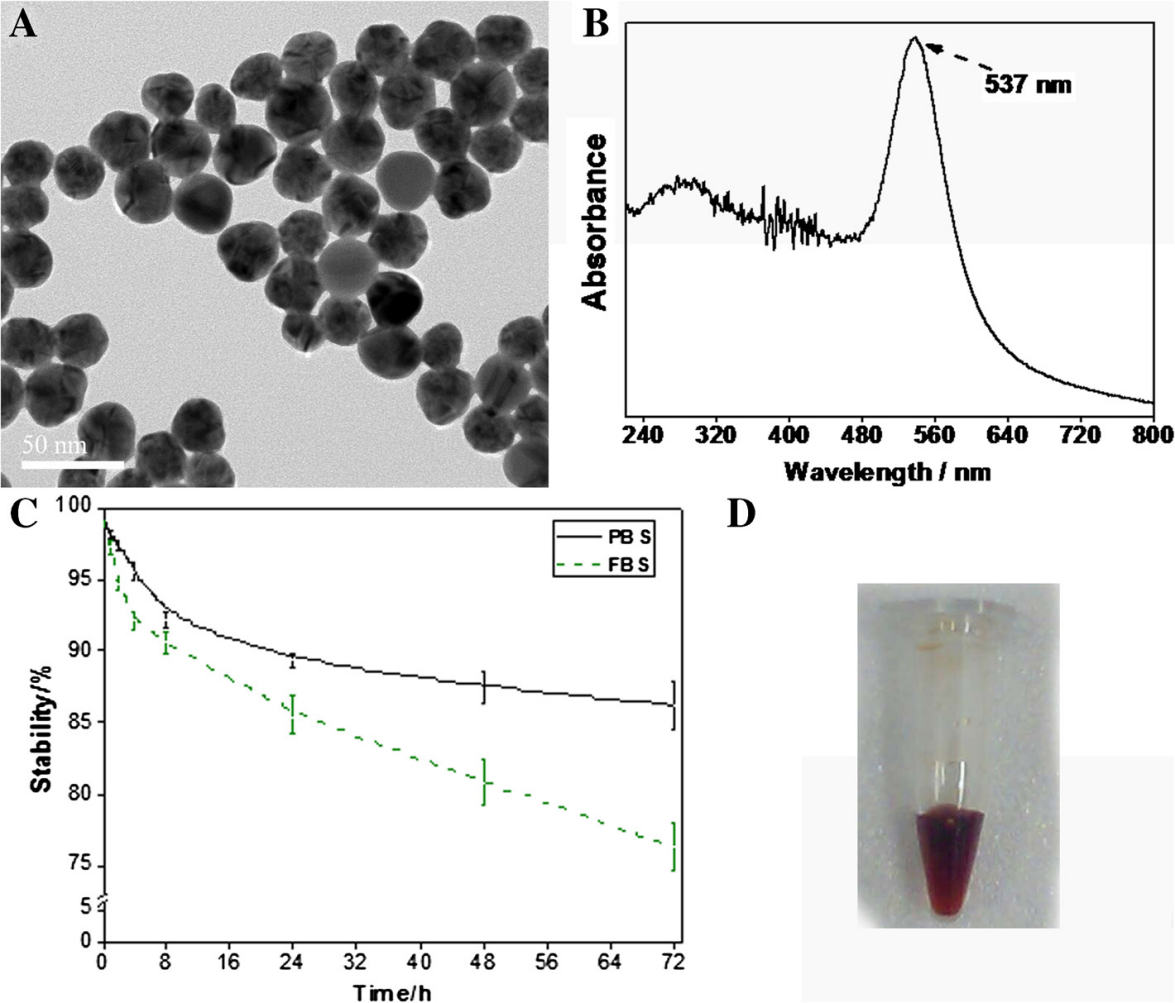
**Characterization of** ^**125**^ **I-cRGD-GNPs. (A)** TEM of nude gold nanoparticles. **(B)** UV spectrum of GNPs. **(C)** *In vitro* stabilities of I-125 labeling in PBS and FBS solution. **(D)** Status of ^125^I-cRGD-GNP injection.

For radionuclide labeling, the labeling rate was 86.3% ± 2.4%; radiochemical purity was more than 99% after centrifugation. I-125-labeled cRGD-GNPs was stable *in vitro*, with a stability of 86.2% ± 1.7% in PBS and 76.3% ± 1.6% in 10% FBS at 3 days after labeling. These guaranteed the long period work of this hybrid nanoparticle system. ^125^I-cRGD-GNPs were water-soluble and wine red. The morphology of GNPs, solution status, and details of stabilities are shown in Figure 2.

### *In vivo* biodistribution

**Figure 3:**
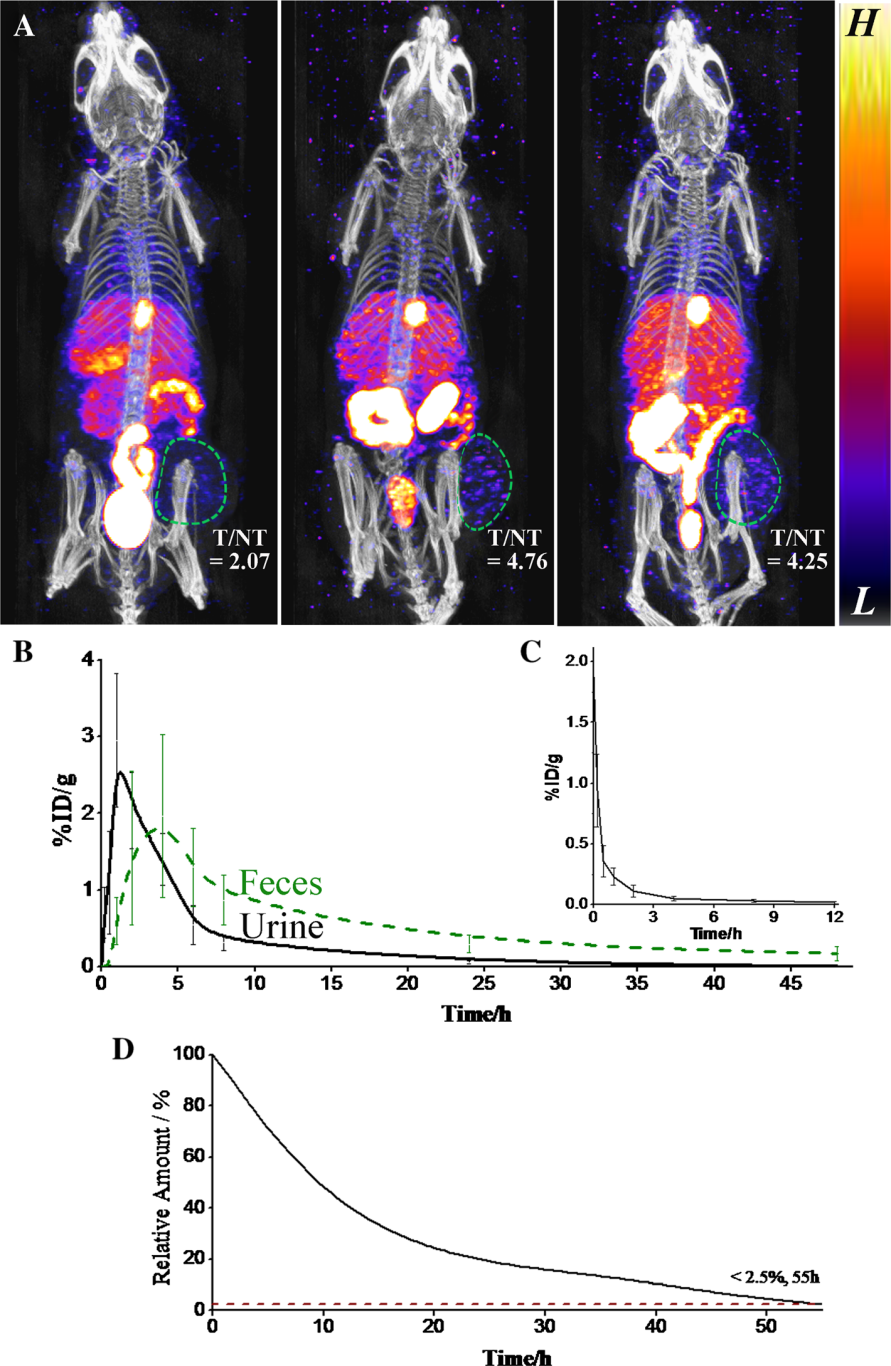
***In vivo*** **mechanism of** ^**125**^ **I-cRGD-GNPs. (A)** SPECT/CT images of tumor-bearing mice at 1, 2, and 4 h post injection; tumor tissues were circled and T/NT values were marked. **(B)** Changes of %ID/g of metabolins from the kidney and intestine. **(C)** Concentration change of radiosensitizer in the blood. **(D)** *In vivo* retained amount of targeted radiosensitizer reflected by retained radioactivity.

*In vivo* biodistribution of cRGD-GNPs was shown by SPECT/CT images (Figure 3A). Attributing to the PEGs covering and cyclic RGD conjugation, cRGD-GNPs targeted tumor more efficiently and avoided heavy capture by the liver, while part of the nanoparticles were still absorbed by the gallbladder. cRGD-GNPs were expelled from the blood system rapidly and targeted tumor with a peak value of T/NT of 4.76 at 2 h post injection. T/NT remained 4.25 at 4 h post injection. cRGD-GNPs were mainly expelled via the kidney during the first 4 h. cRGD-GNPs were also absorbed by the intestine and then expelled gradually during the first 2 days. The detailed changes of radioactivity of the blood, urine, and feces are plotted in Figure 3B, C. Based on the mechanism of ^125^I-cRGD-GNPs, radiotherapy treatments were performed at 4 h post injection with a relatively high T/NT value of 4.25, a precondition of valid radiosensitive RT. Meanwhile, radiosensitized RT-induced damage to normal tissues was controlled to a relatively low level at 4 h post injection. Dynamic monitoring of radioactivity proved that only less than 2.5% radioactivity residual was detected at 55 h post injection (Figure 3D). Herein, assessment of apoptosis based on ^99m^Tc-Annexin V SPECT was performed at 2 days post treatment to reflect the therapeutic effect in acute period.

For tumor-targeted agents, an ideal delivery of nanoparticles to tumor tissues will preferentially increase tumor cytotoxicity and minimize damage to healthy tissues simultaneously. A lot of efforts have been made to facilitate the targeted accumulation of gold nanoparticles, such as the introduction of anti-EGFR antibody [31,32]. Based on a similar mechanism, RGD conjugation approach utilized active angiogenesis targeting as well; furthermore, the rigidity of cyclic RGDyC and unique expression of α_v_β_3_ integrin in blood tumor vessels guarantee the persistence, specificity, and high efficiency of radiosensitizer. On the other side, particle size and surface covering is an important role in determining the clearance. Smaller size and biocompatible materials, such as glutathione [33], are contributing factors in enhancing EPR effect. In this research, functionalized PEG was used and exhibited an appreciating stability and clearance, avoiding abundant uptake by RES. Besides these, the versatility of functionalized PEG provides multiple potential for modification of particles.

### Radiosensitized therapeutic effect

**Figure 4:**
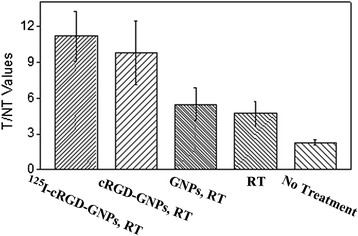
**T/NT values of five groups, indicating the degree of apoptosis.** Significant targeted radiosensitizer-induced apoptosis were observed from the group of ‘^125^I-cRGD-GNPs with RT’ and ‘cRGD-GNPs with RT’.

Apoptosis is one of the main RT-induced responses in early stage. Annexin V specially targets the phosphatidylserine located on the surface of apoptotic tumor cells. Therefore, apoptosis degree detected by ^99m^Tc-Annexin V SPECT closely correlated to the therapeutic effect induced by radiosensitized RT. As reflected by T/NT values (Figure 4), which indicated the degree of apoptosis, therapeutic effects resulting from the introduction of targeted radiosensitizers were more serious than those from radiotherapy alone, as well as the control group, where slight apoptosis resulted from the inflammatory tumor tissues. Although there was not a significant difference between ^125^I-cRGD-GNPs-based RT and cRGD-GNPs-based RT (for T/NT, 11.2 ± 2.1 vs 9.8 ± 2.7, *P* = 0.093), significant differences were exhibited between targeted radiosensitizer-based RT and non-targeted radiosensitizer-based RT (for T/NT, 9.8 ± 2.7 vs 5.5 ± 1.4, *P* = 0.011) and between radiolabeled cRGD-GNPs-based RT and non-targeted radiosensitizer-based RT (for T/NT, 11.2 ± 2.1 vs 5.5 ± 1.4, *P* < 0.01). On the one side, radiosensitive effect were totally shown via ^99m^Tc-Annexin V SPECT for apoptosis; on the other side, targeting effect enhanced radiosensitive effect effectively.

**Figure 5:**
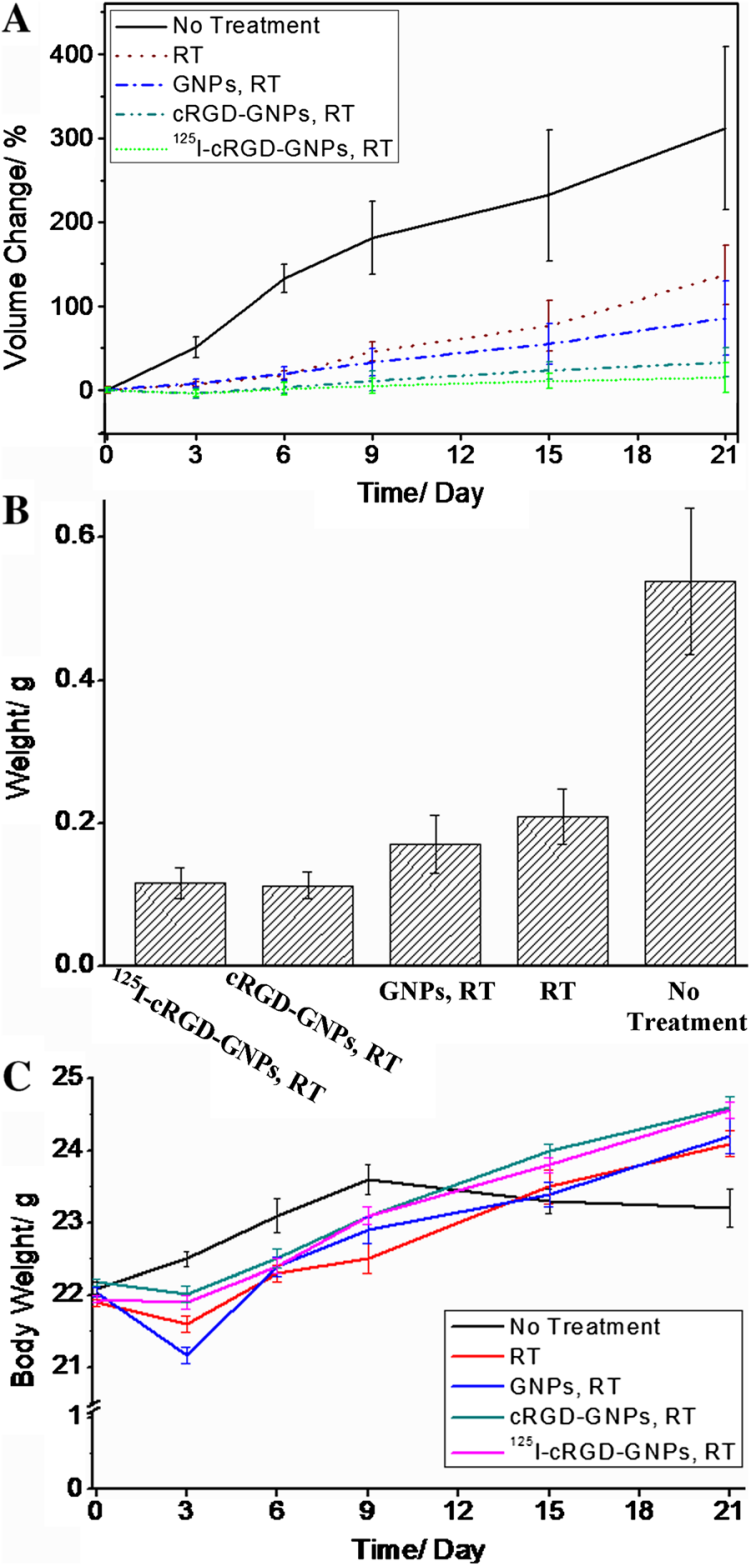
**Long-term evaluation of radiosensitive RT effect. (A)** Changes of tumor volumes at a definite time post injection. **(B)** Weight of excised tumor tissues at the 21st day. **(C)** Body weight changes of tumor-bearing mice.

Long-term observation showed the therapeutic effect of radiosensitized RT directly via tumor volume changes (Figure 5A). On day 21, the increase of tumor volume of mice treated by RT with targeted radiosensitizer was suppressed to 33.1% ± 17.1%. ^125^I-cRGD-GNPs even lead a less increase of 15.2% ± 17.8%. Volume increases of the groups of GNPs with RT, RT alone, and control were 85.5% ± 44.2%, 137.1% ± 35.5%, and 312.1% ± 96.9%, respectively. As shown in Figure 5B, the weight of excised specimen of tumor tissues were 0.116 ± 0.022 (^125^I-cRGD-GNPs, RT), 0.113 ± 0.019 (cRGD-GNPs, RT), 0.171 ± 0.041 (GNPs, RT), 0.209 ± 0.038 (RT only), and 0.538 ± 0.102 g (no treatment), respectively. Additionally, no obvious weight loss of mice was observed (Figure 5C), indicating a low *in vivo* toxicity of PEG-covered GNPs and cRGD-GNPs.

Apoptosis and necrosis are the primary approach of radiotherapy-induced cell death, which related to a series of complex mechanisms, including double-strand break of DNA and mitochondrial damage [34,35]. Apoptosis imaging is an innovative strategy in the pathogenesis detection and early therapeutic effect evaluation, such as tumor response to chemotherapy and radiotherapy [36]. In this research, ^99m^Tc-Annexin V SPECT after radiotherapy, but the commonly used *in vitro* test, such as TUNEL staining and γ-H2AX assay of DNA double-strand break, were utilized, avoiding the separate characterization of apoptosis and long-term therapeutic effect. When considering apoptosis and long-term volume change together, we found that there are a kind of connection and a predicting significance of apoptosis for long-term therapeutic effect. Hence, ^99m^Tc-Annexin V SPECT provides an approach of early evaluation of radiosensitized radiotherapy.

For a better understanding of radiosensitizing effect of GNPs, such as the detailed biodistribution, some efforts have been made emphasizing on the *in vivo* tracking. Different with the introduction of MRI-detectable gadolinium chelates [37], the introduction of I-125 provided a signal closely correlated with the concentration of cRGD-GNPs. Therapeutic effect of I-125 was shown, but not a statistically significant improvement when compared with cRGD-GNP group. Therapeutic effect of radionuclide may be heavily enhanced if a more effective radionuclide, such as I-131, was chosen. On the other hand, based on the point of view that ‘exposure of tumors to irradiation causes transient upregulation of α_v_β_3_ on tumor blood vessel endothelium’ [38,39], cRGD targeting may be enhanced by upregulation of α_v_β_3_ resulting from the introduction of iodine-125 [40]. So, radionuclide plays roles as a radiotracer, a therapeutic factor, as well as a contributing factor for targeting.

## Conclusions

In this research, I-125-labeled cRGD-GNPs were successfully synthesized for targeting tumor tissues. Tumor uptake of cRGD-GNPs reached the peak T/NT value at 2 h post injection. For therapeutic effect, ^125^I-cRGD-GNPs were of a high radiotherapy efficiency due to the radiosensitive property of gold nanoparticles, as well as the introduction of α_v_β_3_-targeted molecule and radionuclide. This study demonstrated that 50-μg GNPs every gram body weight in combination with 5-Gy γ ray radiotherapy at 4 h post injection resulted in effective suppression of tumor growth. These results have important implications in developing radionuclide-labeled radiosensitizer for enhanced radiotherapy.
